# Antimicrobial Properties of the Triclosan-Loaded Polymeric Composite Based on Unsaturated Polyester Resin: Synthesis, Characterization and Activity

**DOI:** 10.3390/polym14040676

**Published:** 2022-02-10

**Authors:** Zhandos Tauanov, Olzhas Zakiruly, Zhuldyz Baimenova, Alzhan Baimenov, Nuraly S. Akimbekov, Dmitriy Berillo

**Affiliations:** 1 Faculty of Chemistry and Chemical Technology, Al-Farabi Kazakh National University, Almaty 050040, Kazakhstan; alzhan.baimenov@nu.edu.kz; 2 Department of Research and Development, LLP “Marmar Kazakhstan”, Taldykorgan 040008, Kazakhstan; zakiruly@gmail.com (O.Z.); zhuldyz.baimenova@gmail.com (Z.B.); 3 Laboratory of Green Energy and Environment, National Laboratory Astana, Nazarbayev University, Nur-Sultan 010000, Kazakhstan; 4 Faculty of Biology and Biotechnology, Al-Farabi Kazakh National University, Almaty 050040, Kazakhstan; Akimbekov.Nuraly@kaznu.kz (N.S.A.); berillo.d@kaznmu.kz (D.B.); 5 Department of Pharmaceutical and Toxicological Chemistry, Pharmacognosy and Botany School of Pharmacy, Asfendiyarov Kazakh National Medical University, Almaty 050000, Kazakhstan

**Keywords:** polymer, composite resin, antimicrobial activity, triclosan, antibacterial

## Abstract

The manufacturing of sanitary and household furniture on a large scale with inherently antimicrobial properties is an essential field of research. This work focuses on the synthesis of polymer composites based on the unsaturated polyester of resin loaded with 5 wt.%-Triclosan produced by a co-mixing approach on automated technological complex with a potential for broad applications. According to findings, the polymer composite has a non-porous structure (surface area < 1.97 m^2^/g) suitable for sanitary applications to reduce the growth of bacteria. The chemical composition confirmed the presence of major elements, and the inclusion of Triclosan was quantitatively confirmed by the appearance of chlorine on XRF (1.67 wt.%) and EDS (1.62 wt.%) analysis. Thermal analysis showed the difference of 5 wt.% in weight loss, which confirms the loading of Triclosan into the polymer matrix. The polymer composite completely inhibited the strains of *S. aureus* 6538-P, *S. aureus* 39, *S. epidermidis* 12228, and *Kl. Pneumoniae* 10031 after 5-min contact time. The antimicrobial effects against *Kl. pneumoniae* 700603, *Ps. aeruginosa* 9027 and *Ps. aeruginosa* TA2 strains were 92.7%, 85.8% and 18.4%, respectively. The inhibition activity against *C. albicans* 10231 and *C. albicans* 2091 was 1.6% and 82.4%, respectively; while the clinical strain of *C. albicans* was inhibited by 92.2%. The polymer composite loaded with 5 wt.%-Triclosan displayed a stability over the period that illustrates the possibility of washing the composite surface.

## 1. Introduction

According to the World Health Organization, dangerous microbes such as bacteria, viruses, and parasites in food cause diarrheal diseases, which make 550 million people sick and cause 230,000 deaths each year, while inadequate drinking water, sanitation, and handwashing practices cause 842,000 diarrheal deaths in low- and middle-income countries. The development of microbial resistance to antibiotics further complicates the situation [1]. Therefore, keeping the sectors of massive contact free of microbial contamination is paramount for the health and safety of people.

Microbial contamination is largely associated with numerous human health-related sectors such as hospitals and dental equipment, food packaging and storage, water treatment systems, kitchen and dining accessories, and household sanitation equipment. The unavoidable presence of harmful microorganisms on the surface of those furniture and daily commodities resulted in the escalation of various infections and diseases. Commonly used surface coatings or backbone materials, such as wood, steel, glass and plastics, could partially “serve” as an environment for the growth and spread of microorganisms [2,3,4]. This might be due to the porous microstructure of those commonly applied porous materials, which allows the penetration of water or moisture to create a potential growth environment for microorganisms [3,4]. Moreover, frequent contact with water or disinfectants due to daily cleaning services could gradually destroy the surface and shorten the duration of kitchen accessories, household and hospital sanitary furniture or wares. To avoid these issues, materials with non-porous structures might be a solution, where either the building block or scaffold itself or the attached bactericidal agent may serve as an inherently antimicrobial material or surface coating for those types of necessities. In addition, the polymeric scaffolds to be used as the backbone material or surface coatings for those applications must have a potential for large-scale manufacturing, as well as to provide materials in different shapes, geometries and sizes to expand the application fields and meet market requirements.

Disinfectants such as hypochlorite, hydrogen peroxide, quaternary ammonium compounds, silver salts, or other reactive oxygen species have been examined for these uses, but their short duration and limited environmental friendliness make their application highly competitive [5]. At the same time, new macromolecules with antimicrobial properties and structural modifications of various materials, including polymers, are being developed to achieve disinfectant properties with desired physicochemical parameters [6]. According to Bonilla et al. [7], antimicrobial materials could be separated into four categories: (a) those that exhibit antimicrobial activity on their own; (b) those for which biocidal activity is obtained by chemical modification; (c) those that include either low or high molecular weight (MW) antimicrobial organic compounds; and (d) those involving the addition of active inorganic systems.

Antimicrobial polymers are a class of materials designed to kill or inhibit the growth of microbes on surfaces or in the environment. Polymers such as chitosan, quaternary nitrogen composites, halamines, and poly-ε-lysine may have inherent antimicrobial properties [8,9], while other polymers act as scaffolds for the immobilization of small biocides and antibiotics to enhance bactericidal activity [10,11,12]. Antimicrobial polymers currently under study include substituted or modified natural polymers, antimicrobial polymers containing multiple biocidal units attached to the backbone, polymers with a satellite terminal biocidal unit, polymer–antibiotic composite, and polymer–inorganic antimicrobial composites [13,14]. Organic (chlorhexidine, triclosan, polyaniline, and polyethyleneimine) agents are commonly used to modify polymers to give them antimicrobial properties. Among these agents, triclosan (2,4,4-trichloro-2-hydroxydiphenyl ether) is widely used in personal care products, such as in toothpaste [15], hand soaps and cosmetics [16], and polymers to prevent the process of degradation and colonization over time [17]. At low concentrations, Triclosan has a bacteriostatic effect, due to the harmful influence of bacterial enzymes responsible for the composition of fatty acids from the cell wall and membrane, while at high concentrations, triclosan destroys the bacterial membrane, leading to its death [18]. Due to its broad antimicrobial spectrum, it is used in commercial resin sealants [19], in halloy–site nanotubes for composite resins [20,21], and has also been encapsulated in nanocapsules for addition to adhesive resin [22], demonstrating good physical and chemical properties without cytotoxicity. However, in studies by Genari et al. [22], it was found that after 120 h, almost 20% of triclosan is released from the adhesive resin network, which might be due to the porous structure of the substrate. Polymeric scaffolds with higher drug retention can reduce the leaching of triclosan and keep its chemical structure available for interaction with bacteria for a prolonged duration. Particularly, the inherently non-porous polymer composites might be a solution, as they entrap the attached antibacterial drugs or agents within the structure of the matrix. Table 1 summarizes a number of related research works on biomedical and antimicrobial applications, where synthetic and natural polymers, as well as polymeric composites employed as support matrices for organic or inorganic antimicrobial agents. However, most of these polymeric substrates have a porous structure to some extent or are limited in processing for small scales only, which may potentially reduce their market application for the manufacturing and production of daily sanitary and household accessories and furniture.

This research work focuses on the synthesis of polymeric composites based on unsaturated polyester resin attached to the organic antimicrobial agent triclosan into non-porous structure by a co-mixing approach on the advanced complex, which allows the manufacturing of polymer composite-based sanitary and household furniture on a large scale. The samples of obtained pristine and triclosan-loaded polymeric composites with antimicrobial properties are characterized on cutting-edge techniques such as XRF, SEM-EDS, CHNS, BET and TGA to retrieve chemical, morphological and thermal characteristics that are essential for the proposed applications. The current research apprehended a broad range of strains of bacteria and fungi that commonly exists on various surfaces in comparison to previous studies. This allows one to effusively evaluate the antimicrobial activity of the pristine and triclosan-loaded polymeric composites. In addition, the mechanical properties of the polymeric composites, antimicrobial performance over several cycles of re-use along with a release of the loaded antimicrobial agent triclosan, have been explored and meticulously discussed.

## 2. Materials and Methods

### 2.1. Materials

The following chemical reagents are used to produce polymer composites with and without loading the antimicrobial agent: unsaturated polyester of orthophthalic acid (99%), methyl ethyl ketone peroxide (99%) and triclosan (5-chloro-2-(2,4-dichloro-phenoxy)phenol) (99.5 wt.%), CaCO_3_ (95 wt.%), lecithin (Phosphatidylcholine: min. 60%, *Escherichia coli:* negative, Iodine number: 60–70; Peroxide number: max. 3.0; Lysophosphatidylcholine: max. 3%; non-polar lipids: max. 20%; Water (K.F.): max. 2%) and tween-80 1310 g/mol were purchased from AppliChem, Darmstadt, Germany. The synthesis of polymer composites with and without loading triclosan was triplicated and average synthesis characteristics are presented. Culture media and reagents nutrient broth, nutrient agar, and tryptic soy broth were purchased from Himedia, Mumbai, India. All chemical reagents were of analytical grade and used as received without prior purification or treatment.

(1) *Staphylococcus aureus* ATCC 6538-P—a sensitive test strain obtained from the Republican collection of microorganisms (RCM), Nur-Sultan, RK;

(2) *Staphylococcus aureus* ATCC BAA-39—a resistant test strain obtained from the American Type Culture Collection (ATCC), Manassas, VA, USA;

(3) *Staphylococcus epidermidis* ATCC 12228—a sensitive test strain obtained from the American Type Culture Collection (ATCC), Manassas, VA, USA;

(4) *Klebsiella pneumoniae* ATCC 10031—a resistant test strain obtained from the American Type Culture Collection (ATCC), Manassas, VA, USA;

(5) *Klebsiella pneumoniae* ATCC 700603—a sensitive test strain obtained from the American Type Culture Collection (ATCC), Manassas, VA, USA;

(6) *Pseudomonas aeruginosa* 9027—a sensitive test strain obtained from the American Type Culture Collection (ATCC), Manassas, VA, USA;

(7) *Pseudomonas aeruginosa* TA2—a resistant clinical isolate;

(8) *Candida albicans* ATCC 10231—a test strain obtained from the American Type Culture Collection (ATCC), Manassas, VA, USA;

(9) *Candida albicans* ATCC 2091—test strain obtained from the American Type Culture Collection (ATCC), Manassas, VA, USA;

(10) *Candida albicans*—a clinical isolate.

### 2.2. Synthesis of Polymer Composites with Triclosan

To produce a composite material, the required amounts of unsaturated polyester resin, filler (CaCO_3_), and additive (Triclosan) were thoroughly mixed in a vacuum mixer until a viscous homogeneous mass was obtained. The ratio of reagents used in the synthesis of polymer composite is shown in Table 2. The presented data show the composition of the reference and experimental samples of polymer composites produced in this research. The experimental sample includes the addition of antimicrobial agent triclosan (5 wt.%), while the reference sample is excluded.

In order to synthesize a non-porous polymeric composite, the liquid substances of the composition unsaturated polyester resins of orthophthalic acid and methyl ethyl ketone peroxide were quickly and vigorously mixed in a preliminary mixer (premixer, RESPECTA, Wulfrath, Germany) with a capacity of at least 2800 rpm within 2 s to avoid the development of bubbles. This is due to the fact that, after the addition of peroxide into the resin, the mixture becomes highly reactive. After this, the mixed liquid substances were further vigorously mixed under 1400 rpm for 5–6 s in the main mixer unit (RESPECTA, Wulfrath, Germany) with the addition of solid components (CaCO_3_ and triclosan). During mixing, a vacuum pump with a power of at least 0.1 bar was used to evacuate air from the mixture. Next, the mixture was poured into special molds, while eliminate the inlet of air from the external environment and inside the mold itself. This was avoided by using closed molds by forcing air out of the mold when the mixture was poured. The rate of the polymerization (hardening) of the material controlled the ambient temperature.

**Figure 1:**
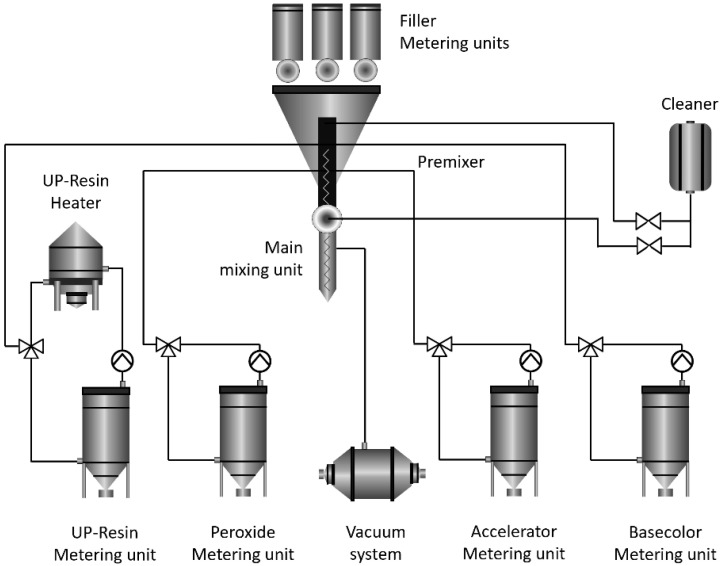
Preparation of polymer composite on automated technological complex.

As the catalyst is added, the resin becomes more viscous, until it reaches a state where it is no longer a liquid and is no longer able to flow. This is the state of termination of polymerization. The resin continues to harden after it has thickened until it reaches its full hardness over time. This reaction itself is accompanied by the release of heat due to an exothermic reaction, which, in turn, accelerates the reaction. The temperature at the peak reaches over 50 °C. The whole process is known as “curing” the resin, which lasts for about 1 h and 20 min. Further details of the synthesis of polymeric composite from unsaturated polyester resin of orthophthalic acid, as well as the alternative synthesis of aromatic copolyesters of biosourced-phthalic acids (dimethoxyterephthalate), tiophene–aromatic polyesters could be found elsewhere [14,39,40]. Figure 1 shows the schematic diagram of the polymer composite production process on an automated technological complex.

### 2.3. Characterization of Polymer Composite

A chemical analysis of polymer composites was conducted using a standardless X-ray fluorescence (XRF, Malvern Panalytical, Cambridge, UK) spectrometer under helium atmosphere in a powder sample mode. For XRF analysis, a tablet was prepared from the following ratio: 5 g of polymer composite and 1.2 g of boric acid additive. The two powders were thoroughly mixed in an agate mortar for 20 min. Then, the fine powder was placed in a steel round mold and placed under a vacuum press at a pressure of at least 20 atm. After 30 min, the tablet was removed and placed in a special plastic sample holder for research. The sample holder was placed in an XRF analyzer and examined for elements in a helium atmosphere. Total carbon, hydrogen, nitrogen, and sulfur elemental analysis was carried out using a vario El Cube (Elementar Analysensysteme, Langenselbold, Germany) CHNS analyzer.

The thermal properties of polymer composite samples were identified using a Simultaneous Thermal Analyzer STA6000 (Perkin Elmer, Waltham, MA, USA). For analysis, about 10 g of a sample was taken and placed in special heat-resistant crucibles. The samples were heated in an atmosphere of pure nitrogen from 50 °C to 700 °C at a heating rate of 10 °C/min to reveal the thermal stability of the polymer composite and measure the amount of inorganic residues (ash) formed after high-temperature combustion.

The morphological characteristics of the polymer composite with and without triclosan were studied on scanning electron microscopy (SEM) using a JEOL 6380LV Scanning Electron Microscope (JEOL, Tokyo, Japan), operating in LV mode, at 20 KV, equipped with a backscattered electron detector. Spot and area analyses were carried out using a Si(Li) Energy-Dispersive X-ray spectrometer (INCA X-sight, Oxford Instruments, Abingdon, UK), connected to SEM.

The porous structure was determined using low-temperature nitrogen adsorption measured on an Autosorb-1 porosimeter (Quantochrome, Hook, UK). The average pore size and total pore volume were calculated from the experimental data obtained from porosimetric studies using the in-built software Asiqwin. The specific surface area was calculated using the BET model. For a functional groups analysis, FT-IR spectra of the samples in the form of fine powder were recorded in the range of 4000–400 cm^−1^ with a resolution of 4 cm^−1^ using a Cary 600 Series FTIR spectrophotometer (Agilent Technologies, Santa Clara, CA, USA) equipped with an ATR module.

### 2.4. Antimicrobial Activity Tests of Polymer Composite

The antimicrobial testing parameters using the pristine and Triclosan-loaded polymer compoistes are shown in Table 3.

#### 2.4.1. Preparation of Test Samples

Before the study, the control and experimental composite samples were placed in individual sterile Petri dishes. At the same time, polyethylene release liner was prepared, the size of which was 40 × 40 (±2.0) mm. Previously, all samples, as well as the release liner, were sterilized with 70% ethanol. After testing each strain, the composite samples were also sterilized with 70% ethanol.

#### 2.4.2. Preparation of Suspensions of Test Strains

Notably, 18–24 h’s culture of test strains were used in the study. In a test tube containing 5–6 mL 1/250 nutrient broth; an aliquot of the corresponding test strain was added with a sterile loop and homogenized. The optical density was measured densitometrically, which was 1.2 units, according to McFarland, which corresponded to a cell concentration of 3.6 × 10^8^ CFU/mL for bacterial suspensions and 3.6 × 10^6^ CFU/mL for yeast cells. The working concentration of cells was prepared by 10-fold serial dilutions of the stock suspension until a final inoculum of 3.6 × 10^5^ CFU/mL was obtained for each test strain.

#### 2.4.3. Inoculation

**Figure 2:**
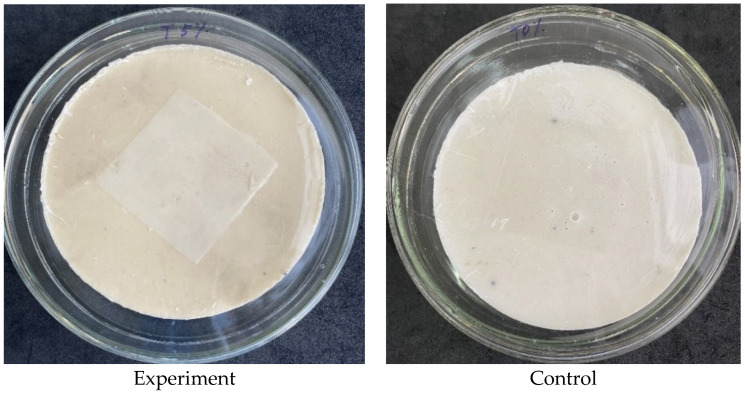
Inoculated experimental (with 5 wt.% Triclosan) and control (without Triclosan) composite samples covered with the release liner.

On the surface of the experimental and control samples, 0.4 mL of the corresponding strain suspension was added. To evenly distribute the inoculum over the surface, the samples were immediately covered with a release liner (Figure 2), after the test samples were placed in a thermostat at 37 °C for two hours of incubation.

#### 2.4.4. Washing Samples with a Neutralizing Agent

After two hours of incubation, the control and experimental samples were washed with a neutralizer as tryptic soy broth with lecithin (1 g/L) and tween-80 (7 g/L) as nontoxic surfactants. Washing was carried out as follows: 10 mL of a neutralizer was added to the Petri dish containing the sample, and the culture was removed from the sample surface, carefully pipetting the contents of the dish.

Note: The use of a neutralizing agent is necessary to avoid false positive results of the antimicrobial activity presence, because the residual amount of the active substance contained in the samples can, to some extent, reduce the number of viable cells.

#### 2.4.5. Inoculation and Counting Colony Forming Units (CFU)

To count CFU, a series of 10-fold serial dilutions (1/10, 1/100) was prepared from the washings of each sample, after which deep inoculation was performed from the washings and prepared dilutions (from one dilution to 2 PE). The amount of inoculum was 1 mL. The inoculated plates were flooded with a warm agar medium (46–48 °C) in a volume of 15–20 mL and the contents were gently mixed. After the solidification of the medium, the dishes were placed in a thermostat. After the incubation time, a direct count of the grown CFUs was performed visually.

#### 2.4.6. Number Determination of the Viable Bacteria

For each sample, the definition of viable bacteria was calculated according to Equation (1):

N = (100 × C × D × V)/A(1)

where,

N is the number of CFU/sample;

C is the average number of CFU on duplicated plates;

D is the dilution factor;

V is the volume of the used neutralizer, mL;

A is the area of the release liner, mm^2^

All experiments were designed and conducted according to standard protocols JIS Z 2801:2000 ISO 22196:2007, ASTM E 1054 [41].

## 3. Results and Discussion

### 3.1. Synthesis of Polymer Composite with Triclosan

The results of the elemental analysis show that the framework of the polymer composite has a carbonate structure, where the content of calcium, as the main mineral, is about 49%, and the remaining elements of the framework of the polymer composite are magnesium, sulfur, iron, oxygen, and carbon. The polymer composite with 5 wt.%-triclosan, in turn, revealed a successful modification with the antibacterial agent triclosan, where 1.67% chlorine is detected, the molecule of which contains three atoms of chlorine (Table 4). These results prove that the polymer composite has been successfully modified with the triclosan and the method are suitable for the synthesis of antibacterial polymer composites.

**Figure 3:**
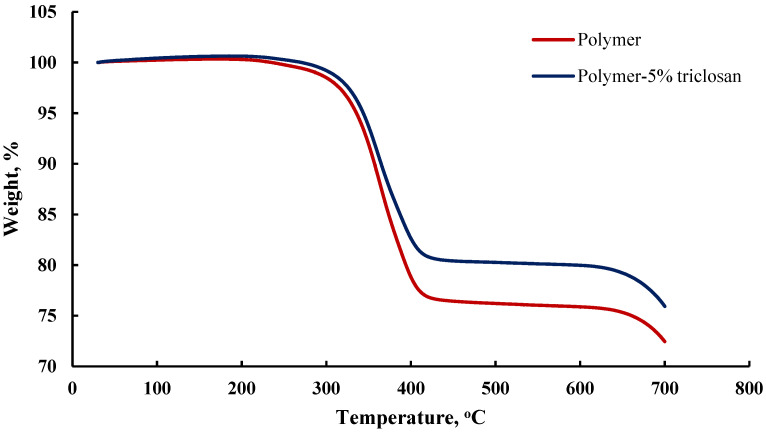
Thermogravimetric analysis of the pristine and 5 wt.%-triclosan-loaded polymer composites at heating rate of 10 °C/min from 50 °C to 700 °C.

It can be concluded that the polymer composite with triclosan has a greater organic component than the polymer composite. It was found that the mass fraction of the combustion residues of the polymer composite after heat treatment in nitrogen flow is about 26%, and for the polymer composite with triclosan, it is about 31%, due to the aromatic nature of the molecule, which also participated in the carbonization process (Figure 3). Moreover, we can confidently assume that the matrix of the polymer composite has a non-porous structure, since, in the case of porous materials, a step of weight loss of adsorbed water should have appeared, due to adsorbed and hygroscopic water in the structure of the material.

**Figure 4:**
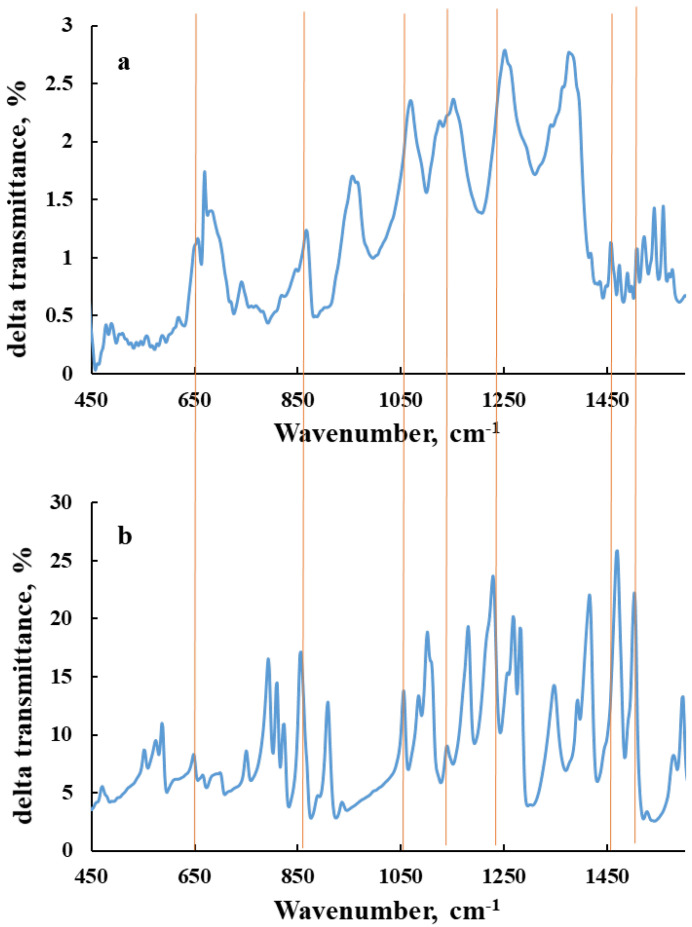
FT-IR spectra of the 5 wt.%-triclosan-loaded polymer composite (**a**) and triclosan (**b**).

The FTIR spectra of polymer composite and 5 wt.%-loaded triclosan polymer composite are presented in Figure 4. Since both samples are made from polymeric precursors, it is very difficult to differentiate the functional groups of triclosan from the polymeric matrix. The peaks at 742 and 871 cm^−1^ are attributed to the =C–H aromatic vibration, and we have polyaromatic polymers that overlay with characteristic bonds of =C–H of triclosan. According to the literature, the high intensity vibration bond for Triclosan is registered at 1485 cm^−1^ Bojar et al. [42]. The frequency C–Cl cannot be detected due to the low content of triclosan in the composition, and due to the average and low intensity of the bond.

**Figure 5:**
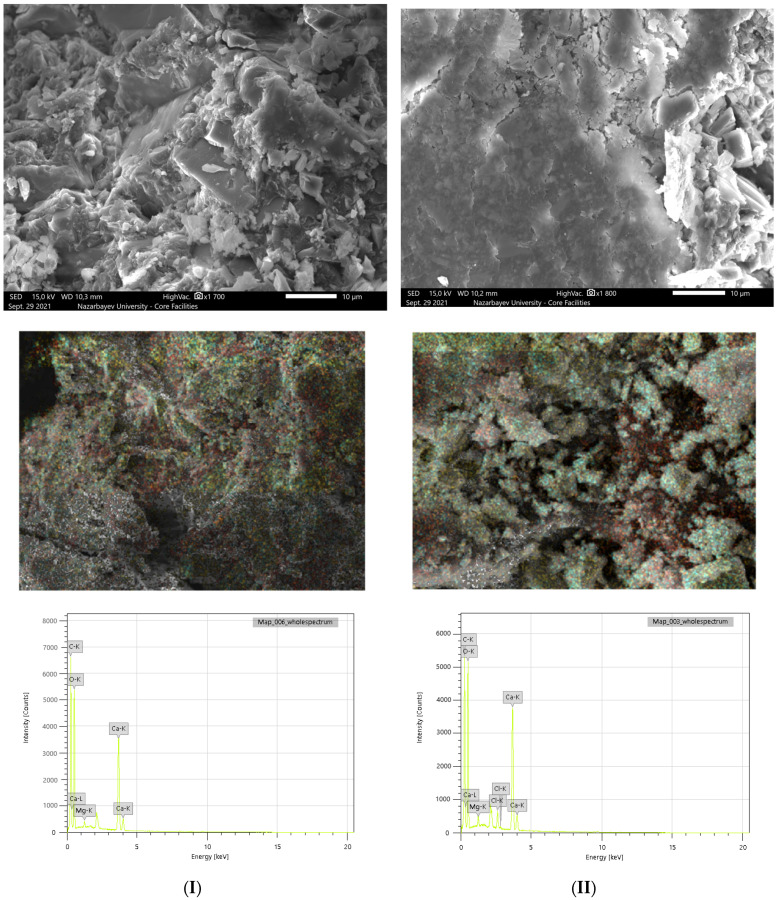
SEM-EDS micrographs of the pristine (**I**) and 5 wt.%-triclosan-loaded (**II**) polymer composites.

The morphological analysis of the original and modified polymer composite with the antibacterial agent triclosan shows the non-porous structure of the samples. These morphological characteristics together with mechanical properties are favorable for the production of sanitary and construction products based on a polymer composite, in order to effectively prevent the spread of microorganisms. The main advantages of the proposed polymer composite are non-porosity (monolithic material without pores) and high maintainability. Due to its non-porosity, microorganisms have less probability to grow on the surface of the material. Therefore, it allows them to be applied as sanitary products and as building materials. In the event of defects on the surface of the material, they can be easily eliminated by sanding and polishing the defective spots. The results of the SEM study of the polymer composite are shown in Figure 5, while the mechanical properties can be found in the Supplementary Materials, Section 1.

Samples of the original and modified polymer composite were also analyzed by the quantitative EDS method, which shows the content of the main elements in the selected area of the SEM micrographs. The mass fractions of calcium and magnesium in the polymer composite are 21.87% and 0.56%, respectively (Table 5). The EDS analysis illustrated that the modified composite, in addition to these metals, revealed 1.92% chlorine, confirming the successful impregnation of triclosan into the matrix of the material, which most probably takes place due to p–p stucking. These results are consistent with those obtained on an X-ray fluorescence analysis, which in turn confirms the validity of the results.

The original polymer composites and polymer composites with the antibacterial agent triclosan (5 wt.%) were studied on a CHNS elemental microanalyzer in duplicate, which allows one to calculate the exact amounts of carbon (C), hydrogen (H), nitrogen (N), and sulfur (S).

The main element in the framework of the samples is carbon, which varies from 24.49 wt.% to 25.95 wt.% (Table 6). Notably, there was a decrease in the mass fraction of nitrogen and sulfur, which is associated with the addition of 5 wt.% of the antibacterial agent tricolsan; each molecule contains three atoms of chlorine. The data obtained are comparable to the results of X-ray fluorescence analysis (XRF) and with an elemental analysis of a scanning electron microscope (SEM-EDS), which also revealed carbon, hydrogen, nitrogen, and sulfur in an approximate mass fraction.

A porosimetric analysis of the pristine and modified polymer composite with 5 wt.%-triclosan shows the insignificant specific surface area for both samples. The specific surface areas of the composite and the modified composite with triclosan were 1.964 m^2^/g and 0 m^2^/g, respectively. A decrease in the specific surface area of the modified polymer composite can be associated with the addition of an antibacterial agent, as a result of which, the structure becomes even denser, and the number of pores is significantly reduced. This is essentially important, as a potential application of the proposed polymer composite with triclosan is the manufacturing of large-scale sanitary and household furniture, where the entrapment of any water or moisture may create an environment for the growth of microorganisms. The pore sizes of the polymer composites, calculated by the BJH method, exhibit a non-porous structure, where the pore radius of the samples is about 17 ± 0.11 Å.

**Figure 6:**
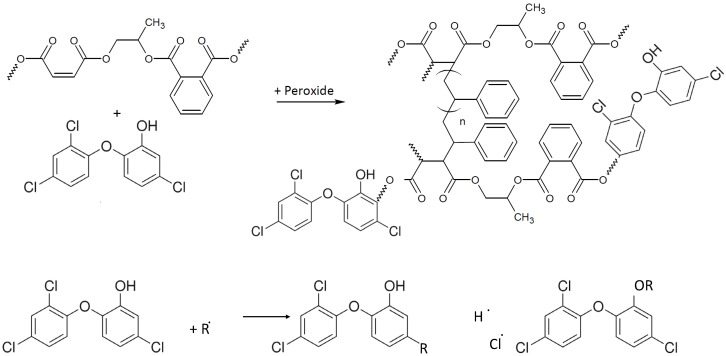
The proposed synthetic reaction of the polymer with triclosan.

A kinetic of release of triclosan from the polymeric composite to water was carried out at room temperature at moderate shaking (160 rpm). No significant increase of the absorbance at 282 nm was observed after five days (Figure S1). From the obtained results, one can conclude that most triclosan is covalently linked to the polymer backbone or it is strongly physically adsorbed on the nonporous polymeric composite via hydrophobic interactions. Moreover, triclosan is a very lipophilic substance with solubility in water at only 10 ppm. It is probable that the accelerated diffusion of triclosan takes place via the lipophilic contact of the cell’s membrane with the composite surface. This finding of triclosan release is very attractive from a practical point of view. It illustrates the possibility of washing the composite surface by pure water with the minimal leakage of the antimicrobial agent, and therefore prolonged usage maintaining a bactericidal effect. Based on the results obtained and the performance of the polymer composite loaded with 5 wt.%-triclosan, the synthetic reaction of polymerization is presented in Figure 6.

### 3.2. Antimicrobial Activity of Polymer Composite with Triclosan

**Figure 7:**
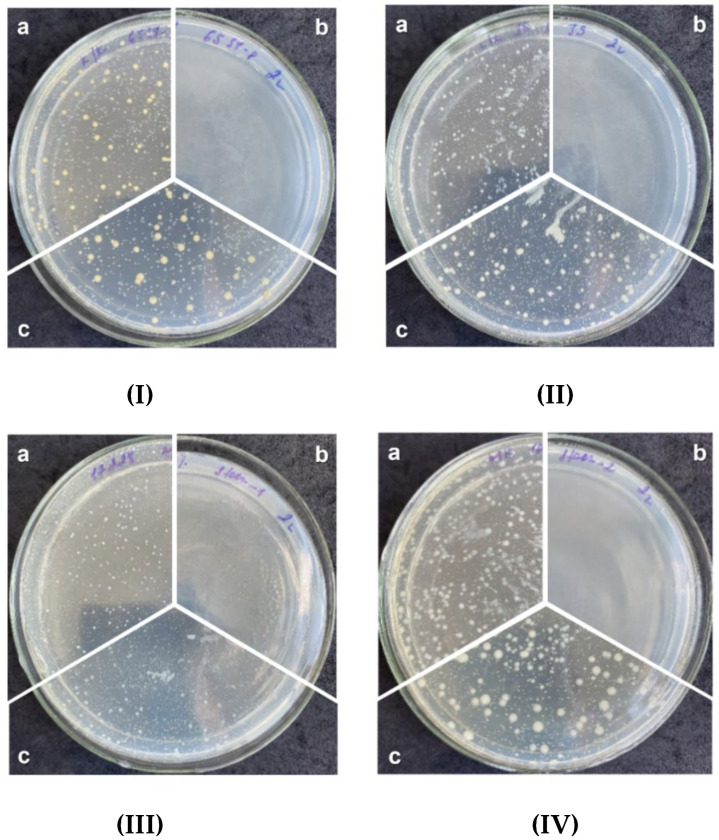
The results of antimicrobial activity testing of composites against *S. aureus* 6538-P (**I**), *S. aureus* 39 (**II**), *S. epidermidis* 12228 (**III**), and *Kl. pneumoniae* 10031 (**IV**): a—negative control; b—Polymer composite with 5 wt.%-Triclosan; c—polymer composite.

**Figure 8:**
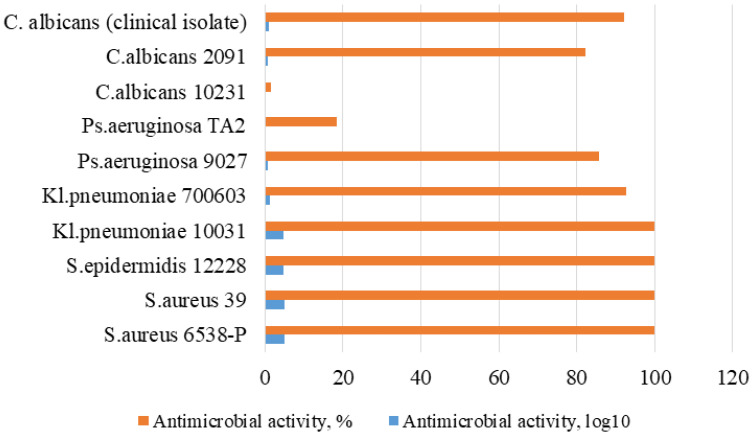
The change in the antimicrobial activity against different strains of microorganisms of the polymer composite with 5 wt.%-triclosan.

The study of the polymeric composite antimicrobial substance triclosan (5 wt.%) was carried out against eight strains of Gram-positive and Gram-negative bacteria at 2-h exposure by direct contact of viable cells of microorganisms with the sample. An identical composite sample without triclosan was used as a negative control. All manipulations with the experimental and control samples were carried out in parallel under the same conditions. The results of antimicrobial activity testing are presented in Table 7 and Figure 7; a comparative graphical interpretation is shown in Figure 8. From previous reports about polymeric composites with triclosan for various composites, we expected that the developed system will illustrate a bacteriostatic effect [43].

The maximum antibacterial effect of Triclosan-containing composite specimens was shown in relation to the strains of *S. aureus* ATSS VAA-39, *S. aureus* ATSS 6538-P, and *S. epidermidis* with aphids, epoxy, epoxy, ATSS, 22x pneumoniae ATSS 10031, which needed only 5 min of contact time with the antimicrobial composite (Figures S2–S4). Some bacteriostatic effect in relation to *P. aeruginosa* ATSS 9027 and fungicidal activity is noted after four hours of contact time with the composite containing triclosan (Figure S5). Thus, as expected, the composite of calcium carbonate, unsaturated ester of orthophthalic acid, and peroxide methyl ethyl ketone did not reveal any bacteriostatic effect.

From the other side, an addition of triclosan (5 wt.%) illustrated significant inhibition of colony growth in vitro. Minimum inhibitory concentrations (MIC) for some strains were higher than for others, which is why longer incubation time with a polymer was necessary to achieve MIC. A high antimicrobial effect against *S. epidermidis* ATSS 12228 and *Kl. pneumoniae* ATSS 10031 at 5 min of contact time (100% cell death) was observed (Table 7). It was reported that the MIC of triclosan against the abovementioned strain is about 1 μg/mL [44], and one can assume that five minutes of incubation provided a diffusion of triclosan which was higher than MIC. The inhibiting activity against *Ps. aeruginosa* ATSS 9027 was noticed only for four hours of incubation (reduction of growth strain by 85.8%) and these data correlate with previously published data illustrating the insignificant activity of triclosan for a concentration range of 12.5–100 μmol/L [45]. The fungicidal activity was estimated against strains of *C. albicans* ATSS 2091 and clinical isolate of *C. albicans* and the reduction of the growth of the strain by 92.2% was at a prolonged contact time of four hours (Table 7). It is worth noting that antimicrobial activity in relation to strains of microorganisms *Kl. pneumoniae* ATSS 700603, *Ps. aeruginosa* TA2 and *C. albicans* ATSS 10231 was not detected (Figures S6–S11). However, according to previous research, the MIC of triclosan for *Kl. pneumoniae* is quite low—about 0.5–0.8 μg/mL [43]. For *C. albicans* ATSS 10231, there is no information for triclosan, but the MIC of caffeine is 12.5 μmol/L [46].

Only five mins of incubation of the triclosan-containing composite sample was enough to observe 100% bactericidal activity against the bacteria of the genus Staphylococcus: *S. aureus* 6538-P, *S. aureus* 39, *S. epidermidis* 12228, as well as the sensitive strain *Kl. pneumoniae* 10031 (Table 7). However, against the resistant strain *Kl. pneumoniae* 700603, the bactericidal activity is reduced to 92.7%, while for *Ps. aeruginosa* 9027 and *Ps. aeruginosa* TA2 strains, it demonstrated bactericidal activity of 85.8% and 18.4%, respectively. The study of fungicidal activity demonstrates a moderate antimicrobial effect against strains *C. albicans* 10231 and *C. albicans* 2091, expressed in 1.6% and 82.4%, respectively, after four hours of contact time. These results are comparable with previous studies conducted with triclosan-loaded chitosan as an antibacterial agent for adhesive resin on the strains of *S. mutans* [23], an antibacterial blend of polyethylene with triclosan on the strains of *E. coli* and *Kl. pneumoniae*; *S. aureus* [24] as well as with the findings on poly(ε-caprolactone)/triclosan-loaded polylactic acid nanoparticles composite examined on the strains of *S. aureus* and *E. coli* [25]. The major advantage of the proposed 5 wt.%-triclosan-loaded polymer composite in the current study is the wide range of studied samples (10 strains), which allowed one to broadly assess the antibacterial performance. Moreover, it was observed that polymer composite reveals a high antimicrobial effect on the clinical strain of *C. albicans* 92.2% (Table 7), which has not been examined on the triclosan-loaded polymer or polymer composite matrix in the literature, to the best of our knowledge.

Thus, the maximum antimicrobial effect of the composite sample with 5 wt.%-triclosan within the studied exposure period was noted against all 10 strains, wherein a complete inhibition was observed for *Staphylococcus aureus* (sensitive and resistant strains) and epidermal staphylococci and susceptible strain *Kl. pneumoniae* 10031 (Figure 7).

**Figure 9:**
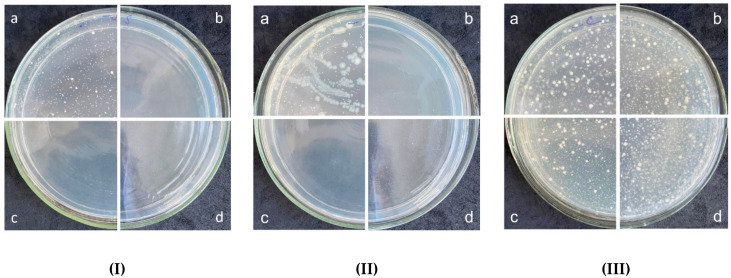
The results of antimicrobial activity of the polymer composite with 5 wt.%-Triclosan against the strain of *S. aureus* 6538-P (**I**), *Kl. pneumoniae* 10031 (**II**), *C. albicans* 10231 (**III**) after three cycles of attrition: a—negative control; b—1st cycle; c—2nd cycle; d—3rd cycle.

Studies to assess the antimicrobial activity of composites under abrasion conditions were carried out against 2 bacterial strains and 1 yeast strain—*S. aureus* ATCC 6538-P, *Kl. pneumoniae* ATCC 10031, *C. albicans* ATCC 10231, respectively. Testing was carried out for three cycles of abrasion. The contact time for bacterial and yeast strains were 2 h and 4 h, respectively. The results of the experiment are presented in Figure 9. The obtained values indicate the preservation of 100% antimicrobial activity against the strain of *S. aureus* 6538-P after three cycles of attrition. The bactericidal activity of the samples against the strain *Kl. pneumoniae* 10031 after three cycles of attrition also remains unchanged. The data obtained when testing the yeast strain *C. albicans* 10231 demonstrate the absence of fungicidal activity after four hours of contact time with the triple abrasion of the samples.

## 4. Conclusions

The polymer composite with antibacterial agent triclosan obtained on an automated technological complex allows one to produce surface coating materials for sanitary applications on a large-scale. According to findings, the polymer composite has indeed a non-porous structure that is further improved by loading antibacterial agent triclosan. The incorporation of triclosan was confirmed by XRF and SEM-EDS analyses, and proved to be a successful modification in terms of the presence of chlorine in the modified polymer substrate. Thermal analysis quantitatively confirmed the difference of 5 wt.% of weight loss between pristine and modified polymer composites, which is in corroboration with the initially loaded triclosan amount that increased aromatic content during carbonization. The antimicrobial activity studies of the triclosan-loaded polymer composite displayed a fast and complete antimicrobial effect against *S. aureus* 6538-P, *S. aureus* 39 strains, *S. epidermidis* 12228, and *Kl. Pneumoniae* 10031. The inhibition performance against remaining strains varied between 1.6% and 92.7%, wherein the antimicrobial activity against a clinical strain of *C. albicans* was 92.2%. The polymer composite showed steady performance after three cycles of use by showing similar antimicrobial activity. The release profile of loaded triclosan within a polymer matrix showed a high stability for a prolonged period, which enhances its potential application as a non-porous building block for a large-scale production of sanitary and household accessories and furniture.

## Figures and Tables

**Table 1:** Selected research on polymer and composite materials loaded with antimicrobial agents.

Title	Support Matrix	Antimicrobial Agent	Antimicrobial Activity Target	Ref.
Triclosan-loaded chitosan as antibacterial agent for adhesive resin	Chitosan	Triclosan	*Streptococcus mutans*	[23]
Preparation and release study of Triclosan in polyethylene/Triclosan anti-bacterial blend	Polyethylene	Triclosan	*Escherichia coli; Klebsiella pneumoniae; Staphylococcus aureus*	[24]
Poly(ε-caprolactone)/Triclosan loaded polylactic acid nanoparticles composite: a longterm antibacterial bionanocomposite with sustained release	Poly(ε-caprolactone)/polylactic acid nanoparticles	Triclosan	*Staphylococcus* *aureus*; *Escherichia coli*	[25]
Antimicrobial polymer composites with copper micro- and nanoparticles: effect of particle size and polymer matrix	Polypropylene; polyamide 6; high-density polyethylene	Copper NPs	*Staphylococcus aureus*; *Pseudomonas aeruginosa*	[26]
Investigations of antibacterial activity of chitosan in the polymeric composite coatings	Chitosan	Acrylic resin	*Staphylococcus aureus*,	[27]
Structural, thermal, and antibacterial properties of chitosan/ZnO composites	Chitosan	Zinc oxide	*Pseudomonas aeruginosa*; *Escherichia coli*; *Staphylococcus aureus*	[28]
Antibacterial effect of composite resins containing quaternary ammonium polyethyleneimine nanoparticles	Restorative composite resin	Quaternary ammonium polyethyleneimine	*Streptococcus mutans*	[29]
Preparation of chitosan-g-poly(acrylamide)/montmorillonite superabsorbent polymer composites: studies on swelling, thermal, and antibacterial properties	Chitosan-g- poly(acrylamide)/montmorillonite	Chitosan	*Staphylococcus aureus*; *Escherichia coli*	[30]
Polyethylene/silver-nanofiber composites: a material for antibacterial films	Polyethylene	Silver nanofiber	*Escherichia coli*	[31]
A novel antibacterial resin composite for improved dental restoratives	Composite resin	Functional furanone derivative	*Streptococcus mutans*	[32]
Structural, thermal and antibacterial properties of polyamide 11/polymeric biocide polyhexamethylene guanidine dodecylbenzenesulfonate composites	Polyamide 11/polymeric biocide polyhexamethylene guanidine	Dodecylbenzenesulfonate	*Escherichia coli*; *Bacillus subtilis*	[33]
Synthesis and characterization of a novel biodegradable antimicrobial polymer	Polyurethane (1,6-hexane diisocyanate, polycaprolactone diol)	Ciprofloxacin (fluoroquinolone antibiotic)	*Pseudomonas aeruginosa*	[34]
Dual-functional antifogging/antimicrobial polymer coating	Poly(2-(dimethylamino)- ethyl methacrylate-co-methyl methacrylate) and polymerized ethylene glycol dimethacrylate network	Quaternary ammonium compound	*Staphylococcus epidermidis; Escherichia coli*	[35]
Antifouling and antimicrobial polymer membranes based on bioinspired polydopamine and strong hydrogen-bonded poly(N-vinyl pyrrolidone)	Polypropylene coated with a polydopamine layer and modified by poly(N-vinyl pyrrolidone)	Iodine	*Staphylococcus aureus*	[36]
Nitric oxide-loaded antimicrobial polymer for the synergistic eradication of bacterial biofilm	Polymer of oligoethylene glycol, hydrophobic ethylhexyl and cationic primary amine	Nitric oxide	*Pseudomonas aeruginosa*	[37]
Dual-mechanism antimicrobial polymer–ZnO nanoparticle and crystal violet-encapsulated silicone	Medical grade silicone	Di(octyl)- phosphinic acid capped ZnO nanoparticles	*Staphylococcus aureus*; *Escherichia coli*	[11]
Novel antimicrobial polymer films active against bacteria and fungi	Polypropylene and linear low-density polyethylene	4′-hydroxy-(4- hydroxy-3-ethyl)-azobenzene (azo-dye)	*Staphilococcus aureus*; *Candida albicans*	[38]

**Table 2:** Production of polymer composite loaded with triclosan.

Sample Name	Composition of Samples	Weight %
Reference polymer composite	Unsaturated polyester resin	28
	Calcium carbonate	70
	Methyl ethyl ketone peroxide (MEKP)	2
Triclosan loaded polymer composite	Unsaturated polyester resin	28
	Calcium carbonate	65
	Methyl ethyl ketone peroxide (MEKP)	2
	Triclosan	5

**Table 3:** The parameters used in the biological studies.

Surface Type of Sample	Chemical Composition				
Sample	calcium carbonate, unsaturated ester of orthophthalic acid, methyl ethyl ketone peroxide, triclosan content 5 wt.%; 8 cm in diameter				
Reference sample	calcium carbonate, unsaturated ester of orthophthalic acid, methyl ethyl ketone peroxide without triclosan; 8 cm in diameter				
Release liner size	40 mm × 40 mm				
Test strains cultivation Medium	Nutrient agar, pH 7.4 ± 0.2	Incubation time and conditions	37 ± 1 °C; 18–24 h		
Inoculum preparation medium	1/250 Nutrient broth, pH 7.4 ± 0.2	Inoculum concentration	2.5–10.0 × 10^5^ CFU/mL	Amount of applied inoculum	0.4 mL
Contact time and incubation conditions	37 ± 1 °C; Humidity: ≥90%, 2 h				
Neutralizer	Tryptic soy broth with lecithin and tween-80, pH 6.8–7.2				
Medium for counting CFU	Nutrient agar, pH 7.4 ± 0.2	Incubation time and conditions	37 ± 1 °C; 40–48 h		

**Table 4:** Chemical composition of polymer composites (%).

Chemical Elements	Content in Polymer Composite	Polymer Composite with 5 wt.%-Triclosan
Mg	0.40	0.39
Ca	48.99	47.61
S	0.02	0.02
Fe	0.01	0.01
Cl	-	1.67

**Table 5:** The elemental analysis of the polymeric composites on SEM-EDS (in wt.%).

Sample	Ca	Mg	C	O	Cl
Polymer composite	21.87	0.56	32.00	45.57	-
Polymer composite with 5 wt.%-triclosan	22.89	0.77	29.97	44.45	1.92

**Table 6:** The microelemental analysis of polymeric composites on a CHNS analyzer (in wt.%).

Sample	C	H	N	S
Polymer composite	25.95 ± 0.34	1.14 ± 0.15	4.99 ± 0.71	0.32 ± 0.05
Polymer composite with 5 wt.%-triclosan	24.49 ± 0.52	1.24 ± 0.02	2.36 ± 0.45	0.28 ± 0.01

**Table 7:** Results of dynamics of antimicrobial activity of the polymer composite with triclosan.

Strain	Contact Time	Sample	Average CFU	Average log10	Antimicrobial Activity, log10	Antimicrobial Activity,%
*S. aureus* 6538-P	5 min	negative control	7.50 × 10^3^	3.875	5.079	100
		composite triclosan	0.06	−1.204		
*S. aureus* 39	5 min	negative control	6.38 × 10^3^	3.804	5.009	100
		composite triclosan	0.06	−1.204		
*S. epidermidis* 12228	5 min	negative control	4.16 × 10^3^	3.619	4.823	100
		composite triclosan	0.06	−1.204		
*Kl. pneumoniae* 10031	5 min	negative control	2.91 × 10^3^	3.463	4.667	100
		composite triclosan	0.06	−1.204		
*Kl. pneumoniae* 700603	5 min	negative control	8.88 × 10^3^	3.948	0.005	1.1
		composite triclosan	8.78 × 10^3^	3.944		
	15 min	negative control	9.06 × 10^3^	3.957	0.006	1.4
		composite triclosan	8.94 × 10^3^	3.951		
	30 min	negative control	7.19 × 10^3^	3.857	0.006	1.3
		composite triclosan	7.09 × 10^3^	3.851		
	1 h	negative control	7.16 × 10^3^	3.855	0.006	1.3
		composite triclosan	7.06 × 10^3^	3.849		
	2 h	negative control	6.19 × 10^3^	3.792	0.104	21.2
		composite triclosan	4.88 × 10^3^	3.688		
	4 h	negative control	2.05 × 10^4^	4.311	1.135	92.7
		composite triclosan	1.50 × 10^3^	3.176		
*Ps. aeruginosa* 9027	5 min	negative control	8.34 × 10^3^	3.921	0.002	0.4
		composite triclosan	8.31 × 10^3^	3.920		
	15 min	negative control	6.66 × 10^3^	3.823	0.002	0.5
		composite triclosan	6.73 × 10^3^	3.821		
	30 min	negative control	6.94 × 10^3^	3.841	0.004	0.9
		composite triclosan	6.88 × 10^3^	3.837		
	1 h	negative control	6.69 × 10^3^	8.825	0.004	0.9
		composite triclosan	6.63 × 10^3^	3.821		
	2 h	negative control	5.72 × 10^3^	3.757	0.005	1.1
		composite triclosan	5.66 × 10^3^	3.753		
	4 h	negative control	3.31 × 10^3^	3.520	0.849	85.8
		composite triclosan	0.47 × 10^3^	2.671		
*Ps. aeruginosa* TA2	5 min	negative control	2.66 × 10^3^	3.424	0.016	3.5
		composite triclosan	2.56 × 10^3^	3.409		
	15 min	negative control	2.84 × 10^3^	3.454	0.020	4.4
		composite triclosan	2.72 × 10^3^	3.434		
	30 min	negative control	3.00 × 10^3^	3.477	0.018	4.2
		composite triclosan	2.88 × 10^3^	3.459		
	1 h	negative control	1.66 × 10^3^	3.219	0.034	7.5
		composite triclosan	1.53 × 10^3^	3.185		
	2 h	negative control	1.22 × 10^3^	3.086	0.086	17.9
		composite triclosan	1.00 × 10^3^	3.00		
	4 h	Negative control	1.19 × 10^3^	3.075	0.088	18.4
		Composite triclosan	0.97 × 10^3^	2.986		
*C. albicans* 10231	5 min	Negative control	1.12 × 10^4^	4.049	0.001	0.3
		Composite triclosan	1.12 × 10^4^	4.048		
	15 min	Negative control	1.08 × 10^4^	4.034	0.001	0.3
		Composite triclosan	1.08 × 10^4^	4.033		
	30 min	Negative control	9.16 × 10^3^	3.962	0.001	0.3
		Composite triclosan	9.13 × 10^3^	3.960		
	1 h	Negative control	3.38 × 10^3^	3.972	0.004	1.0
		Composite triclosan	3.97 × 10^3^	3.968		
	2 h	Negative control	1.08 × 10^4^	4.035	0.006	1.4
		Composite triclosan	1.07 × 10^4^	4.029		
	4 h	Negative control	9.97 × 10^3^	3.999	0.007	1.6
		Composite triclosan	9.81 × 10^3^	3.992		
*C. albicans* 2091	5 min	Negative control	8.19 × 10^3^	3.913	0.002	0.4
		Composite triclosan	8.16 × 10^3^	3.911		
	15 min	Negative control	5.28 × 10^3^	3.723	0.003	0.6
		Composite triclosan	5.25 × 10^3^	3.720		
	30 min	Negative control	5.53 × 10^3^	3.743	0.002	0.6
		Composite triclosan	5.50 × 10^3^	3.740		
	1 h	Negative control	5.12 × 10^3^	3.710	0.003	0.6
		Composite triclosan	5.09 × 10^3^	3.707		
	2 h	Negative control	5.09 × 10^3^	3.707	0.051	11.0
		Composite triclosan	4.53 × 10^3^	3.656		
	4 h	Negative control	3.84 × 10^3^	3.585	0.747	82.1
		Composite triclosan	0.69 × 10^3^	2.837		
*C. albicans* (clinical isolate)	5 min	Negative control	8.38 × 10^3^	3.923	0.002	0.4
		Composite triclosan	8.34 × 10^3^	3.921		
	15 min	Negative control	8.78 × 10^3^	3.944	0.002	0.4
		Composite triclosan	8.75 × 10^3^	3.942		
	30 min	Negative control	8.63 × 10^3^	3.936	0.003	0.7
		Composite triclosan	8.56 × 10^3^	3.933		
	1 h	Negative control	8.97 × 10^3^	3.953	0.181	34.1
		Composite triclosan	5.91 × 10^3^	3.771		
	2 h	Negative control	1.67 × 10^4^	4.222	0.352	55.5
		Composite triclosan	7.41 × 10^3^	3.870		
	4 h	Negative control	8.84 × 10^3^	3.947	1.109	92.2
		Composite triclosan	0.69 × 10^3^	2.837		

## Data Availability

The data presented in this study are available in Supplementary Material.

## Supplementary Materials

The following are available online at https://www.mdpi.com/article/10.3390/polym14040676/s1, Figure S1: UV-Vis spectrum of the release kinetics of Triclosan from polymer matrix (triplicated), Figure S2: The results of antimicrobial activity of composites against strain *S. aureus* 6538-P contact time 5 min, Figure S3: The results of antimicrobial activity of composites against strain *S. aureus* 39 contact time 5 min, Figure S4. The results of antimicrobial activity of composites against strain *S. epidermidis* 12228 contact time 5 min, Figure S5: The results of antimicrobial activity of composites against strain *Ps. aeruginosa* 9027, Figure S6: The results of antimicrobial activity of composites against strain *Kl. pneumoniae* 700603, Figure S7: The results of antimicrobial activity of composites against strain *C. albicans* 10231, contact time 2 h, Figure S8: The results of antimicrobial activity of composites against strain *C. albicans* 2091, contact time 2 h, Figure S9: The results of antimicrobial activity of composites against strain *C. albicans* contact time 2 h, Figure S10: The results of antimicrobial activity of composites against strain *Kl. pneumoniae* 10031 contact time 5 min, Figure S11: The results of antimicrobial activity of composites against strain *Kl. pneumoniae* 700603, contact time 2 h.
