# Supplementary material for: Antimicrobial Properties of the Triclosan-Loaded Polymeric Composite Based on Unsaturated Polyester Resin: Synthesis, Characterization and Activity
Source: Polymers (Basel). 2022 Feb 10;14(4):676. doi: 10.3390/polym14040676 (PMC8875966; doi:10.3390/polym14040676)
Supplement: Supplementary file 1 [file polymers-14-00676-s001.zip › polymers-1582744-supplementary.pdf]

# Supplementary material

# Triclosan-loaded Polymer Composite with Antimicrobial Properties: Synthesis, Characterization and Antimicrobial Testing

**Z. Tauanov[1,2 *], O. Zakiruly[2], Z. Baimenova[2], A. Baimenov[1], N.S. Akimbekov[3], D. Berillo[3,4]**

[1] Faculty of Chemistry and Chemical Technology, al-Farabi Kazakh National University, Almaty, Kazakhstan

[2] LLP "Marmar Kazakhstan", Taldykorgan, Kazakhstan

[3] Faculty of Biology and Biotechnology, al-Farabi Kazakh National University, Almaty, Kazakhstan

[4] Department of Pharmaceutical and Toxicological chemistry, Pharmacognosy and botany School of Pharmacy at Asfendiyarov Kazakh National Medical University,

* Corresponding author: zhtauanov@nu.edu.kz; tauanov.zhandos@gmail.com

## 1. Physical and mechanical properties of polymer composite

General properties: Density 2000-2200 kg/m$^3$, strength on compression 700-1200 MPa, strength on bending 24 MPa, strength on elongation 16-20 MPa, abrasion 0.02-0.03 g/cm$^2$, coefficient thermal conductivity 0.05-0.85 W/(mC), thermal expansion coefficient 0.013-0.025 mm, elasticity 30000-40000 MPa.

Other physical and mechanical properties of polymer composite:
- Bending strength: 59-63 MPa;
- Module for bending: 9.4-10.5 GPa;
- Temperature of thermal deformation: 98-105 ° C;
- Hardness on Barkol: 68-70;
- Resilience: no damage, (fall
measure weighing 220 g with a height of 92 cm);
- Density: 2000-2200 kg / m3;
- Strength at compression: 100-1200 MPa;
- Erasure: 0.02-0.03 g / cm2;
- Coefficient of thermal conductivity: 0.05-0.85W / (mK);
- Coefficient of thermal expansion: 0.013-0.025 mm;
- Flexibility: 10000-40000 MPa;
- Water absorption: 0.05-0.1%.

2. **The Triclosan release kinetics after 5 days from the polymer matrix**

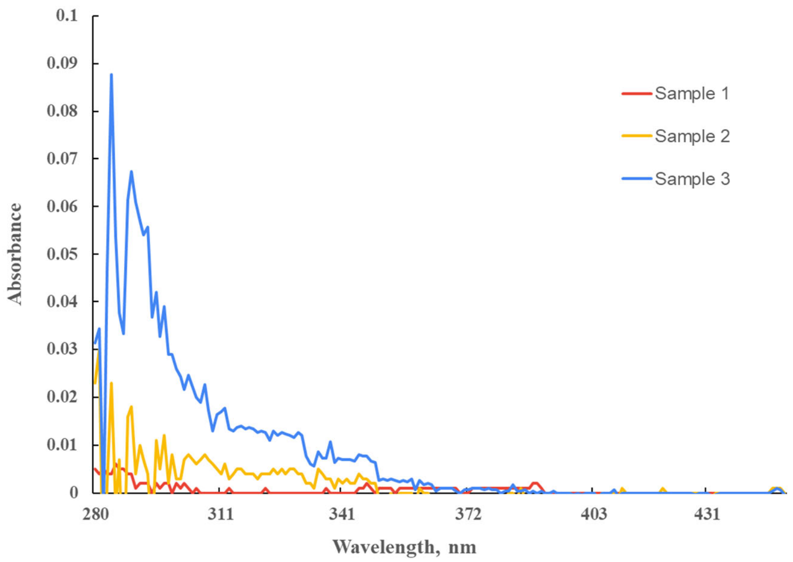


Figure S1. UV-Vis spectrum of the release kinetics of Triclosan from polymer matrix (triplicated)

3. **Antimicrobial testing results**

4\. 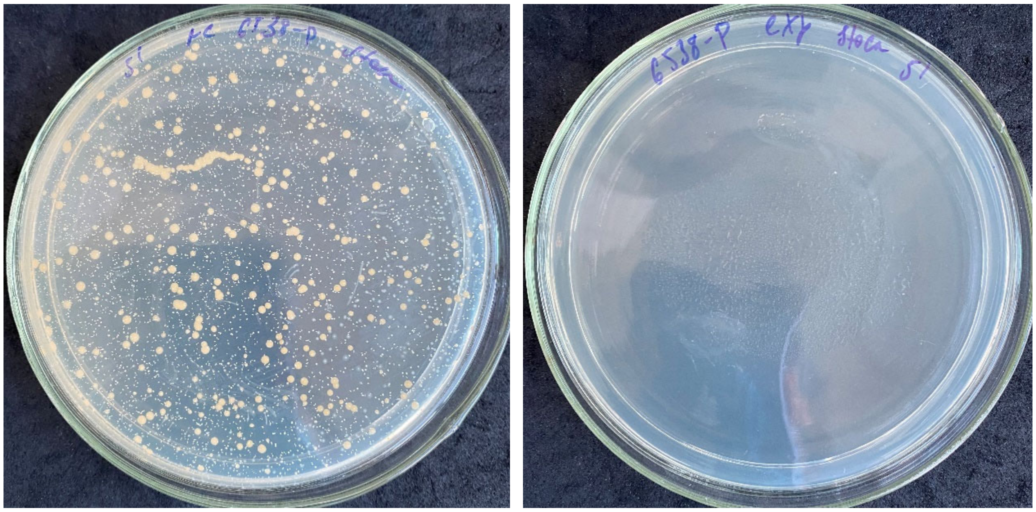

Polymer composite without triclosan

Polymer composite with 5% triclosan

Figure S2. The results of antimicrobial activity of composites against strain *S.aureus* 6538-P contact time 5 minutes

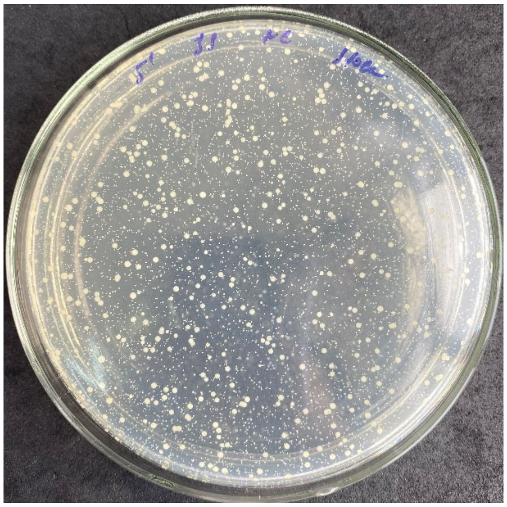

Polymer composite without triclosan

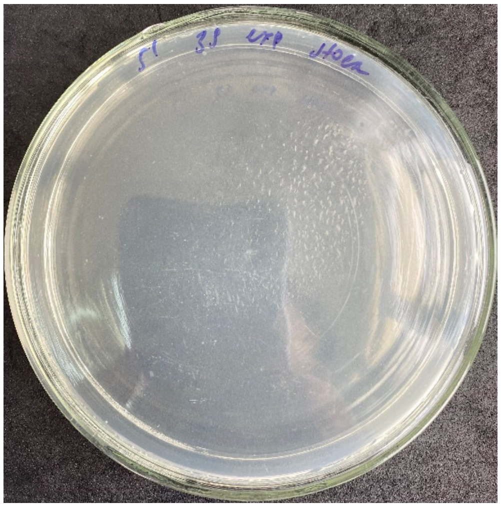

Polymer composite with

5% triclosan

Figure S3. The results of antimicrobial activity of composites against strain *S.aureus* 39 contact time 5 minutes

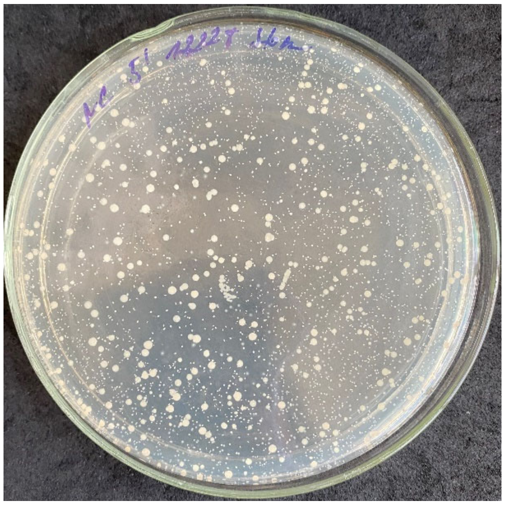

Polymer composite without triclosan

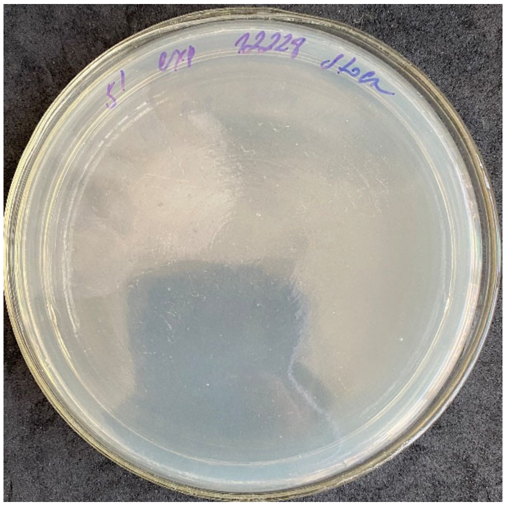

Polymer composite with

5% triclosan

Figure S4. The results of antimicrobial activity of composites against strain *S.epidermidis 12228* contact time 5 minutes

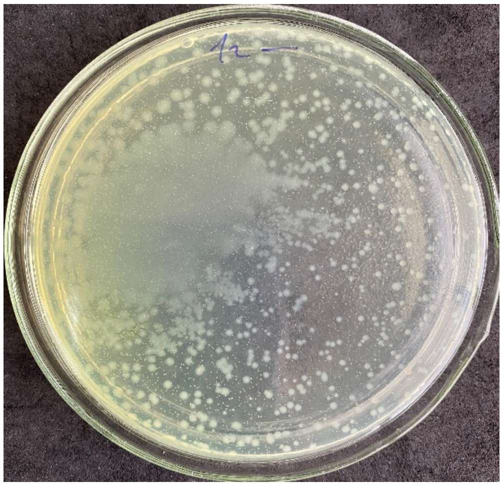

Polymer composite  without triclosan,
contact time 1h

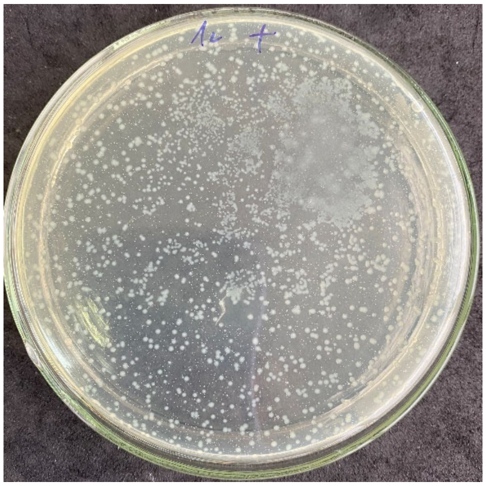

Polymer composite with 5% triclosan,
contact time 1h

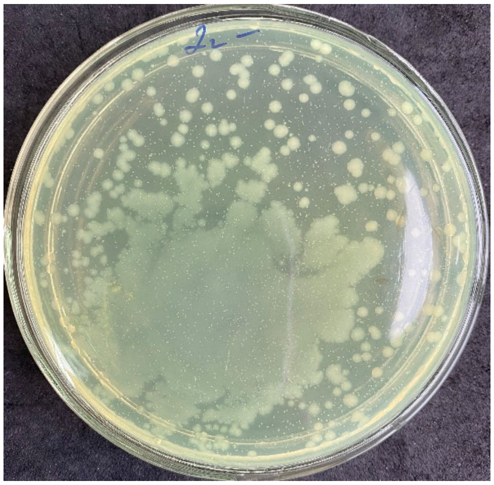

Polymer composite  without triclosan,
contact time 2h

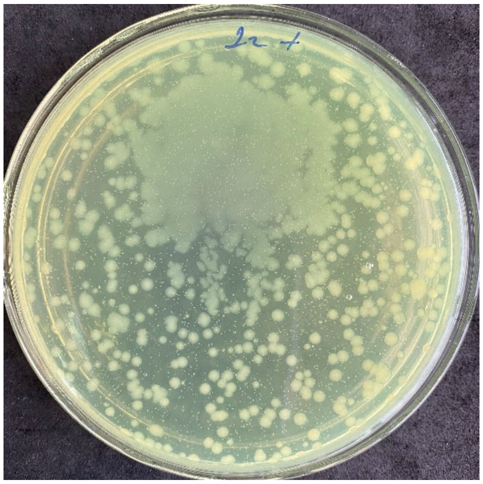

Polymer composite with 5% triclosan,
contact time 2h

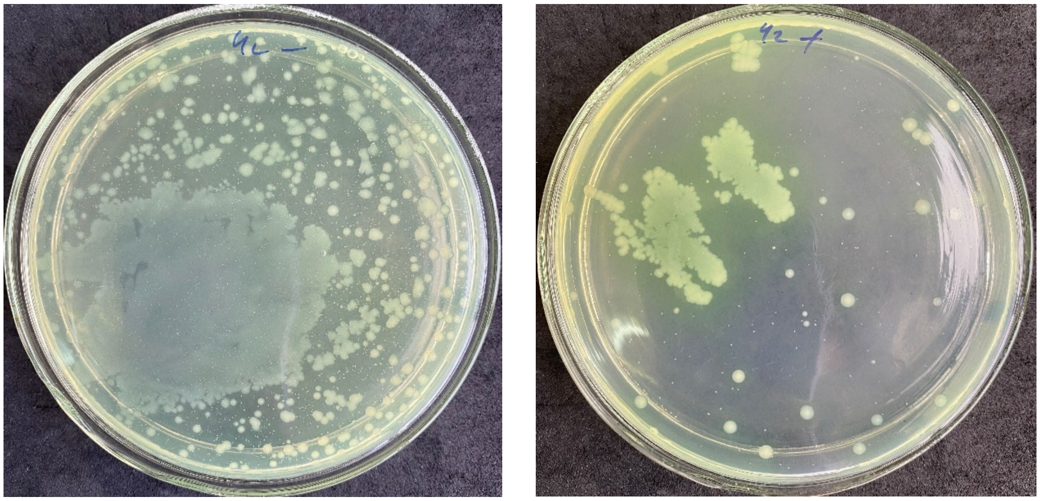

Polymer composite without triclosan, contact time 4h

Polymer composite with 5% triclosan, contact time 4h

Figure S5. The results of antimicrobial activity of composites against strain *Ps.aeruginosa* 9027

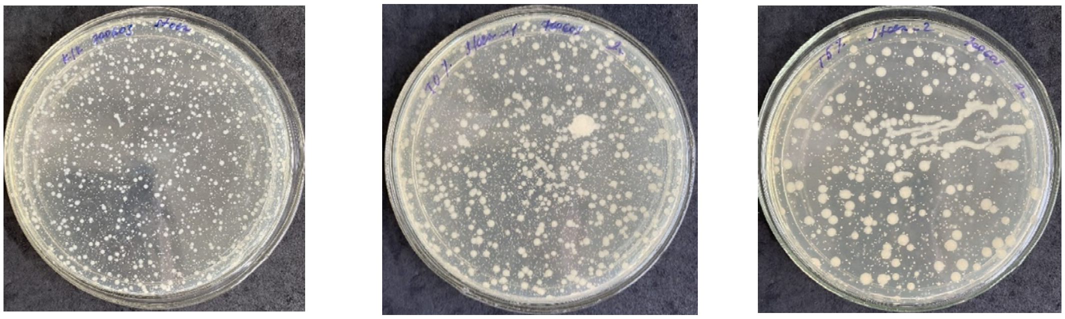

Culture control

Negative control (no triclosan)

Polymer composite with 5% triclosan

Figure S6. The results of antimicrobial activity of composites against strain *Kl.pneumoniae 700603, contact time 2h*

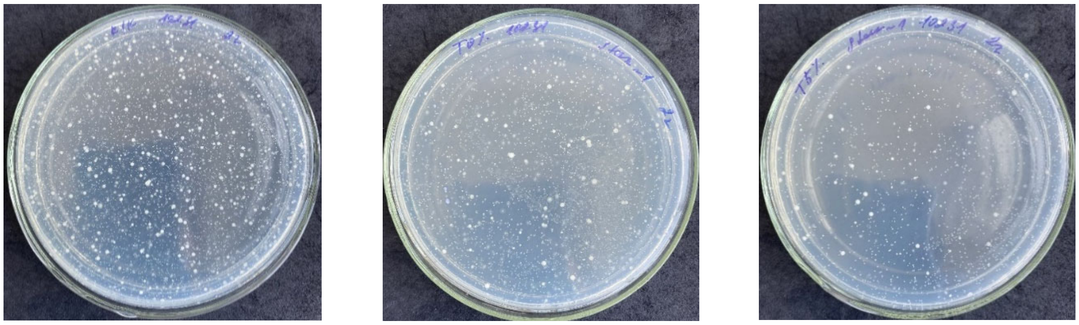

Culture control

Negative control (no triclosan)

Polymer composite with 5% triclosan

Figure S7. The results of antimicrobial activity of composites against strain *C.albicans 10231, contact time 2h*

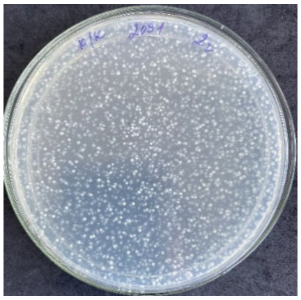

Negative control

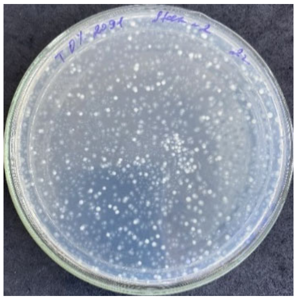

Polymer composite without triclosan

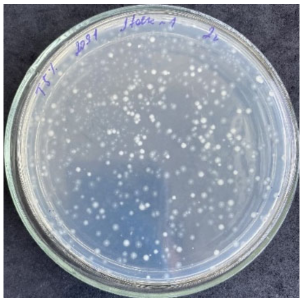

Polymer composite with

5% triclosan

Figure S8. The results of antimicrobial activity of composites against strain *C.albicans 2091,* contact time 2h

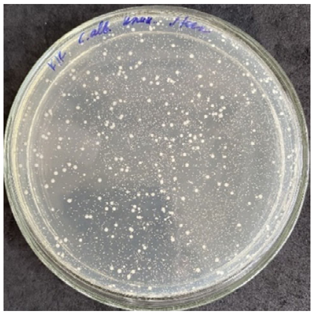

Negative control

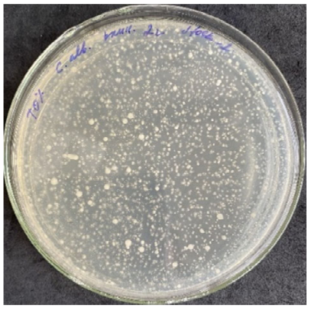

Polymer composite without triclosan

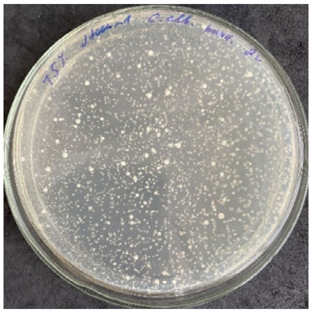

Polymer composite with

5% triclosan

Figure S9. The results of antimicrobial activity of composites against strain *C.albicans* contact time 2h

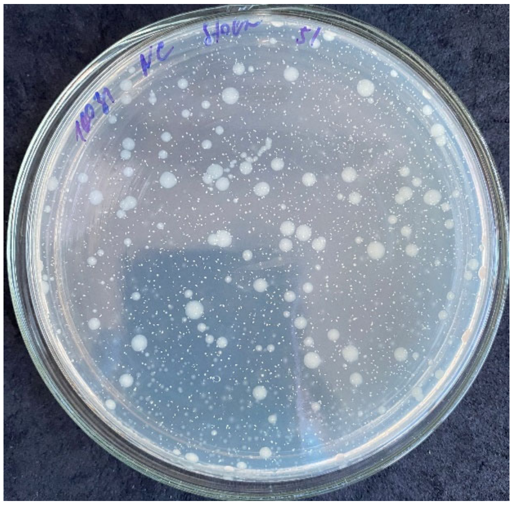

Polymer composite  without triclosan

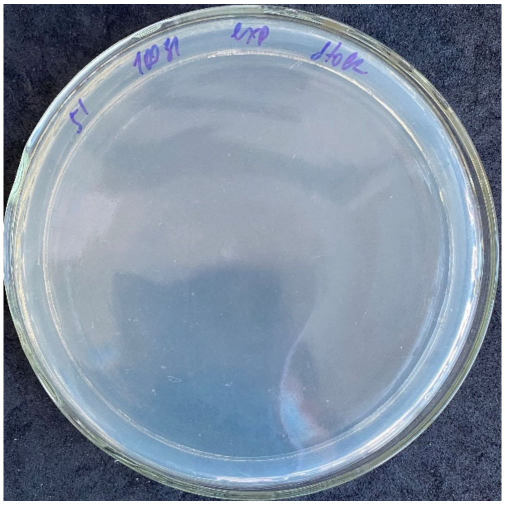

Polymer composite with

5% triclosan

Figure S10. The results of antimicrobial activity of composites against strain *Kl.pneumoniae 10031* contact time 5 minutes

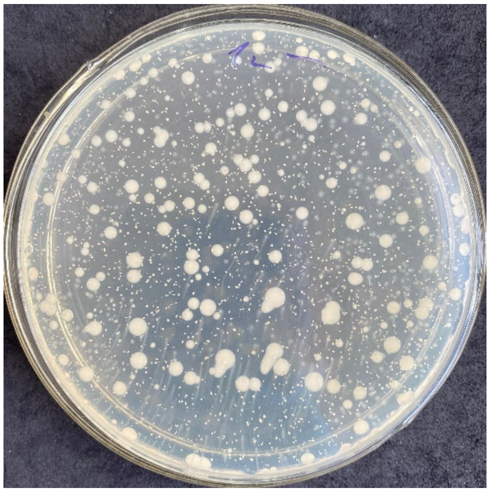

Polymer composite  without triclosan
contact time 1h

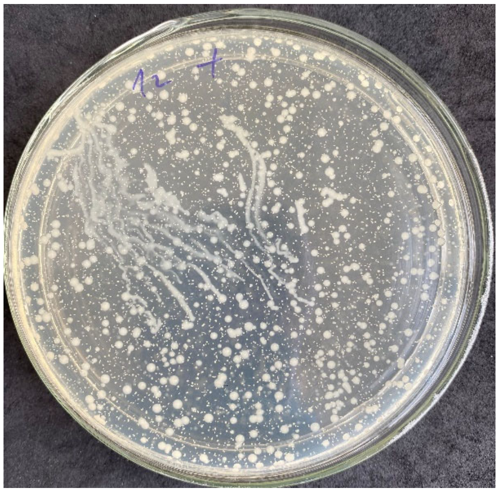

Polymer composite with

5% triclosan contact time 1h

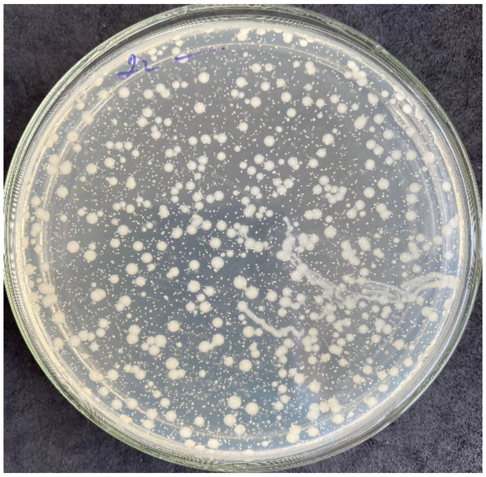

Polymer composite  without triclosan
contact time 2h

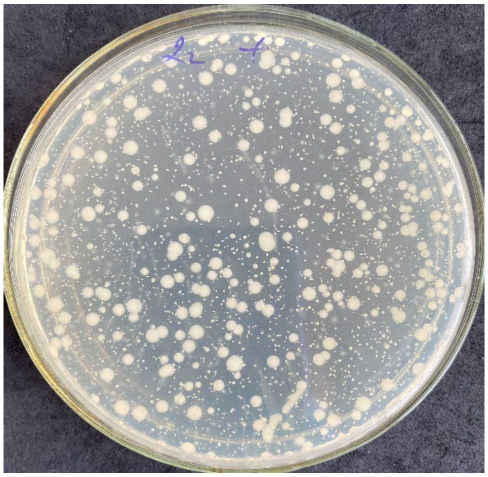

Polymer composite with 5% triclosan
contact time 2h

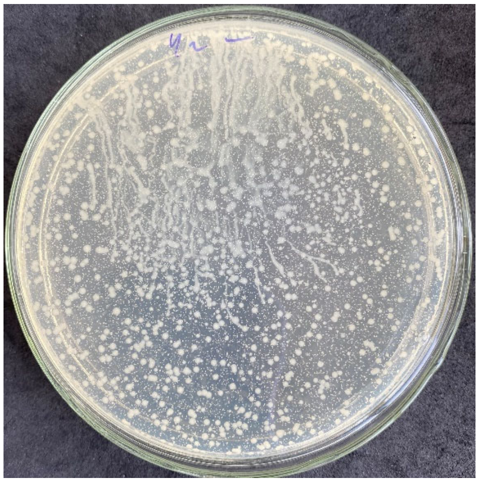

Polymer composite  without triclosan,
contact time 4h

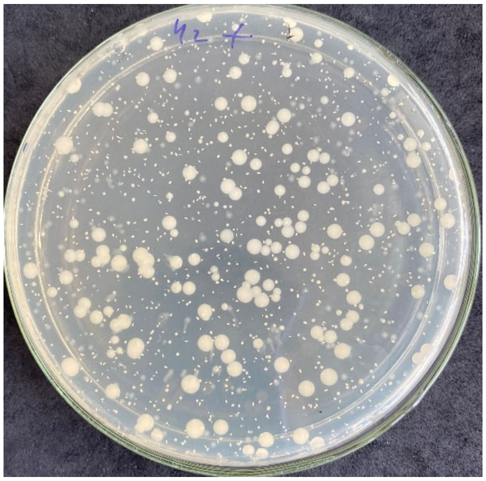

Polymer composite with

5% triclosan, contact time 4h

Figure S11. The results of antimicrobial activity of composites against strain *Kl.pneumoniae 700603*

**References:**

1) JIS Z 2801:2000 − Antimicrobial products-Test for antimicrobial activity and efficacy
2) SO 22196:2007 – Plastics - Measurement of antibacterial activity on plastics surfaces
3) ASTM E 1054 − Standard Practices for Evaluation of Inactivators of Antimicrobial Agents

4) Cornelia Wiegand, Andrea Volpel, Andrea Ewald, et.al. Critical physiological factors influencing the outcome of antimicrobial testing according to ISO 22196 / JIS Z 2801, PLoS ONE 13(3): e0194339. https://doi.org/10.1371/journal.pone.0194339
